# Integrating the RE-AIM Framework and Theory of Change in the Evaluation of Health Interventions: Scoping Review

**DOI:** 10.2196/95809

**Published:** 2026-11-11

**Authors:** Daniela Batista Leite, Gabrielli Barbosa de Carvalho, Marcele Stephanie de Souza Buto, Michelle Louvaes Garcia, Alberto Martins de Jesus, Rafael de Albuquerque e Silva, Roberta R de Lima, Maria C C L B de Andrade, Giovanni Guido Cerri, Carlos Roberto Ribeiro de Carvalho, Paulo Schor

**Affiliations:** 1 Núcleo de Ciências Sociais Aplicadas (NCSA), Universidade de Mogi das Cruzes (UMC), Mogi das Cruzes, São Paulo, Brazil; 2 Laboratório de Genômica, Núcleo Integrado de Biotecnologia (NIB), Universidade de Mogi das Cruzes (UMC), Mogi das Cruzes, São Paulo, Brazil; 3 Programa de Pesquisa, Desenvolvimento e Inovação (PDI) em Saúde Digital, Hospital das Clínicas (HCFMUSP), Faculdade de Medicina (FMUSP), Universidade de São Paulo (USP), São Paulo, Brazil; 4 Coordenação de Saúde Digital, Secretaria de Estado da Saúde de São Paulo (SES/SP), São Paulo, Brazil; 5 Instituto de Radiologia (InRad), Hospital das Clínicas (HCFMUSP); Faculdade de Medicina (FMUSP), Universidade de São Paulo (USP), São Paulo, Brazil; 6 Saúde Digital, Núcleo de Inovação Tecnológica (InovaHC), Hospital das Clínicas (HCFMUSP); Faculdade de Medicina (FMUSP), Universidade de São Paulo (USP), São Paulo, Brazil; 7 Divisão de Pneumologia, Instituto do Coração (InCor), Hospital das Clínicas (HCFMUSP); Faculdade de Medicina (FMUSP), Universidade de São Paulo (USP), São Paulo, Brazil; 8 Departamento de Oftalmologia e Ciências Visuais – Escola Paulista de Medicina, Universidade Federal de São Paulo (UNIFESP), Rua Botucatu, 740, São Paulo, 04023-062, Brazil, 55 11991343694

**Keywords:** reach, effectiveness, adoption, implementation, and maintenance, RE-AIM, theory of change, logic models, implementation science, health interventions, digital health, telehealth, scoping review

## Abstract

**Background:**

Frameworks for implementation evaluation and program theory have been increasingly used to support the design, implementation, and assessment of complex health interventions. The reach, effectiveness, adoption, implementation, and maintenance (RE-AIM) framework provides a structured approach to evaluating reach, effectiveness, adoption, implementation, and maintenance, while the theory of change and logic models explicitly articulate causal mechanisms and pathways of impact. Despite their conceptual complementarity, how these approaches have been jointly applied in empirical studies, particularly in digital health and telehealth contexts, remains insufficiently mapped.

**Objective:**

This scoping review aimed to map and characterize how empirical studies have integrated the RE-AIM framework with theory of change or logic models in the design, implementation, and evaluation of health interventions; identify levels and patterns of integration; and examine how this combined use has been applied in digital health and telehealth interventions.

**Methods:**

A scoping review was conducted following the methodological guidance of the Joanna Briggs Institute and reported in accordance with the PRISMA-ScR (Preferred Reporting Items for Systematic Reviews and Meta-Analyses Extension for Scoping Reviews) checklist. Searches were performed in the PubMed (MEDLINE), Embase, Scopus, and Web of Science databases without restrictions on language, study design, or publication year. Eligible sources were studies, protocols, implementation reports, and other indexed publications that explicitly addressed both the RE-AIM framework and theory of change or logic models in the context of health interventions. Data were extracted using a structured chart and analyzed descriptively to identify characteristics of the included studies, levels of framework integration, and patterns of use across intervention types.

**Results:**

A total of 28 studies published between 2016 and 2025 met the inclusion criteria. Digital health or telehealth constituted a core component in 21.4% (n=6), was present in a limited or secondary role in 21.4% (n=6), and was not identified in 57.1% (n=16) of the studies. Strong integration between the RE-AIM framework and theory of change or logic models was identified in 57.1% (n=16) of the studies, moderate integration was identified in 35.7% (n=10), and weak integration was identified in 3.6% (n=1); integration was classified as not applicable in 3.6% (n=1) of the studies, which were of a methodological and conceptual nature.

**Conclusions:**

The integration of the RE-AIM framework with theory of change or logic models represents a promising methodological strategy for strengthening the planning, implementation, and evaluation of complex health interventions, including digital health initiatives. While the combined use of these frameworks has increased over time, important gaps persist, particularly regarding long-term sustainability assessment and systematic application in telehealth and digital health contexts. Greater methodological rigor and deeper integration between causal models and implementation metrics are needed to advance the field.

## Introduction

### Background

The implementation and evaluation of health interventions are central to strengthening health systems. Health service delivery has become more complex with the expansion of digital technologies and growing demands for effectiveness, equity, and long-term sustainability at the population level. Over recent decades, implementation frameworks have been developed to support this process. Among these, the reach, effectiveness, adoption, implementation, and maintenance (RE-AIM) framework is one of the most widely used approaches for planning and evaluating health programs, with the explicit aim of maximizing public health impact and emphasizing real-world applicability rather than short-term study outcomes [1,2].

In parallel, theory of change and logic models have been increasingly used in health program evaluation to articulate causal mechanisms, assumptions, and pathways linking interventions to intended outcomes. By making these relationships explicit, these approaches support transparency and analytical rigor in both planning and evaluation and clarify how and why interventions are expected to produce effects within specific contexts [3-5]. In this review, theory of change is used as the broader concept for articulating how and why an intervention is expected to produce change, while logic models are considered structured representations of these pathways; specific implementation-oriented logic models such as the implementation research logic model are treated within the latter category [3,4,6].

### Prior Work and Rationale

The convergence of implementation frameworks and causal program theories represents a promising but still insufficiently systematized methodological development. The RE-AIM framework provides a pragmatic structure for assessing reach, adoption, implementation fidelity, and sustainability, whereas theory of change and logic models facilitate deeper examination of mechanisms, contextual determinants, and intervention pathways. Together, these approaches have the potential to strengthen the design, refinement, and evaluation of complex health interventions [7], particularly in areas such as digital health and telehealth, where service models, technologies, and infrastructures have evolved rapidly [8].

Despite this potential, there remains limited clarity regarding how the RE-AIM framework and theory of change have been combined in research. Studies vary considerably in terms of which RE-AIM domains are operationalized, how causal mechanisms are represented in logic models, and whether the two frameworks are meaningfully connected during implementation and evaluation [9,10]. Previous studies have examined the use of the RE-AIM framework or logic models separately, but their combined application has not been systematically characterized, particularly in interventions involving digital health technologies [11]. Gaps therefore remain regarding patterns of use and strategies for methodological integration between these frameworks [12].

### Study Objectives and Research Questions

Given the conceptual heterogeneity and methodological diversity of this field, a scoping review is well suited to map existing evidence, organize the available literature, and identify knowledge gaps without restricting study designs or levels of evidence, in line with guidance from the Joanna Briggs Institute (JBI) [13].

Accordingly, the objective of this scoping review was to examine how scientific literature has integrated the RE-AIM framework with theory of change or logic models in the implementation and evaluation of health interventions, including digital health and telehealth programs.

This review addresses the following research questions (RQs):

1. How have the RE-AIM framework and theory of change or logic models been combined in the design, implementation, and evaluation of health interventions?
2. Which levels and patterns of integration between these frameworks can be identified in the literature?
3. How has this integrated use been applied in digital health and telehealth interventions, and which gaps remain?

## Methods

### Protocol and Guidance

This scoping review was conducted in accordance with the methodological guidance of the JBI for scoping reviews, which emphasizes the systematic identification, mapping, and characterization of evidence without applying restrictive eligibility criteria or formal methodological quality appraisal [13]. Reporting followed the PRISMA-ScR (Preferred Reporting Items for Systematic Reviews and Meta-Analyses Extension for Scoping Reviews) checklist to ensure transparency and completeness of reporting [14] (Checklist 1). The study selection process was documented in a PRISMA-ScR flow diagram, including the number of records identified, screened, excluded, and included in the review. The research team has established expertise in implementation science, program evaluation, and digital health.

### Eligibility Criteria

Publications were eligible if they addressed the use or integration of the RE-AIM framework and theory of change or logic models in the design, implementation, or evaluation of health interventions. Studies of any design were eligible, including quantitative, qualitative, and mixed methods studies; trials; quasi-experimental studies; process evaluations; feasibility studies; and protocols, consistent with JBI guidance for scoping reviews [13]. Methodological or conceptual articles were also eligible when they addressed the relationship between the RE-AIM framework and theory of change or logic models in the context of health interventions. Digital health and telehealth interventions were considered a specific context of interest but were not required for inclusion. Preprints and clinical trial registry entries without associated publications were excluded. Conference abstracts indexed in the databases searched were eligible when they provided sufficient information to confirm the use of both the RE-AIM framework and theory of change or logic models.

### Search Strategy

The search strategy was designed to identify studies that explicitly articulated the RE-AIM framework with theory of change or logic models in the context of health interventions. The RE-AIM framework was originally developed to assess the public health impact of interventions across the dimensions of reach, effectiveness, adoption, implementation, and maintenance [1,9]. Theory of change and logic models were included as complementary approaches aimed at making causal mechanisms, assumptions, and pathways of change explicit within complex interventions [3,4].

The search also encompassed interventions involving digital health, including telehealth, telemedicine, eHealth, and mobile health in accordance with World Health Organization classifications [8]. Controlled vocabulary and free-text terms were combined and adapted to the syntax and indexing characteristics of each database.

Four databases with broad coverage of public health, implementation research, and health interventions were searched: PubMed (MEDLINE), Embase, Scopus, and Web of Science. The complementary coverage of these databases has been documented in comparative indexing studies [15]. Table 1 summarizes the core search concepts and terms, while the complete search strategies as executed in each database are provided in Multimedia Appendix 1. Searches were completed in December 2025.

**Table 1.** Core concepts and search terms.

Search operator	Framework	Conceptual approach	Intervention context
AND (main search term)	RE-AIM^a^	Theory of Change / Logic Model	Health interventions
OR (synonyms)	“RE-AIM” OR REAIM	“theory of change” OR “logic model” OR “program theory”	health OR telehealth OR telemedicine OR eHealth OR mHealth OR “digital health”

a RE-AIM: reach, effectiveness, adoption, implementation, and maintenance.

### Conceptual Framework Alignment

Theory of change and logic models were treated as conceptually equivalent categories, consistent with classic program evaluation literature in which logic models are understood as structured and operational representations of an underlying theory of change [3,4]. In contemporary implementation research, terminology such as “logic model,” “program logic,” and “implementation research logic model” is more commonly used than “theory of change” despite fulfilling the same explanatory function of articulating causal pathways, assumptions, and mechanisms [2,6]. Considering these approaches jointly was therefore methodologically justified and aligned with prevailing usage in scientific literature. Key terms and operational definitions used in this review are provided in Multimedia Appendix 2.

### Study Selection

All records retrieved from the database searches were imported into Zotero (version 7.0; Corporation for Digital Scholarship) [16] for reference management. Duplicate records were removed automatically and verified manually to identify overlapping entries across databases and multiple publications related to the same intervention.

Title and abstract screening and eligibility assessment of the retrieved publications were conducted manually by 2 reviewers. Discrepancies were resolved through consensus with a third reviewer. In line with methodological guidance for scoping reviews, no formal interrater reliability statistics were calculated as the objective was to map the extent and characteristics of the literature rather than appraise study quality [14].

### Data Extraction and Analysis

Data were extracted into a structured spreadsheet capturing bibliographic information, methodological characteristics, type of intervention, thematic area, identification of digital health and telehealth components, modes of RE-AIM application, approaches to the use of theory of change or logic models, and a narrative synthesis of relevant findings. Digital health was considered the broader category, with telehealth representing a specific modality involving the remote delivery or support of health services. Digital health and telehealth components were coded as “yes,” “partial,” or “no,” where “yes” indicated that a digital health or telehealth component constituted a core element of the intervention, “partial” indicated that such a component was present but played a limited or secondary role, and “no” indicated that no digital health or telehealth component was identified.

To characterize the level of integration between the RE-AIM framework and theory of change or logic models, an analytic qualitative classification was conducted based on explicit, predefined criteria. Studies were classified as demonstrating strong integration when the RE-AIM framework and theory of change or logic models were jointly and explicitly used to inform intervention design, implementation, and evaluation, with clear articulation between causal mechanisms and evaluative domains. Studies were classified as demonstrating moderate integration when theory of change or logic models were used primarily for planning or conceptual framing and the RE-AIM framework was applied mainly as an evaluative structure without systematic linkage between causal assumptions and outcome measurement. Weak integration was defined as the parallel or descriptive use of both frameworks without explicit articulation between proposed mechanisms and RE-AIM dimensions. The classification was conducted by 2 reviewers based on the information available in the included publications and the predefined criteria described above. Disagreements were resolved through consensus with a third reviewer.

Analysis was conducted descriptively, with a focus on identifying patterns of framework use, levels and forms of integration, and applications across intervention contexts, as well as gaps in the literature and opportunities for methodological integration between implementation evaluation and programmatic models. In accordance with JBI recommendations, no assessment of risk of bias or methodological quality appraisal was performed as these procedures are not required for scoping reviews [13]. The complete data extraction chart is provided in Multimedia Appendix 3 [17-44].

In addition to domain-level coding and classification of framework integration, a cross-study analytic synthesis was performed to identify recurring patterns across the included literature. This synthesis was based on comparative analysis of extracted variables, including year of publication, intervention context, level of RE-AIM–theory of change integration, presence of digital or telehealth components, and RE-AIM domain coverage. Patterns were identified through iterative team discussion and consensus, consistent with the descriptive and exploratory aims of scoping reviews.

## Results

### Overview

A total of 63 records were identified through searches conducted in the PubMed, Embase, Scopus, and Web of Science databases. After removal of 23 duplicates, 40 records remained for title screening. Following title and abstract screening, 30 records were assessed for eligibility. Two publications were excluded at this stage because they did not explicitly meet the eligibility criterion requiring use of both the RE-AIM framework and theory of change or programmatic logic models. Consequently, 28 publications met the eligibility criteria and were included in this scoping review.

The unit of analysis was the individual included publication. The study selection process is shown in Figure 1, and the detailed data extraction chart for the included studies is provided in Multimedia Appendix 3 [17-44].

**Figure 1.**
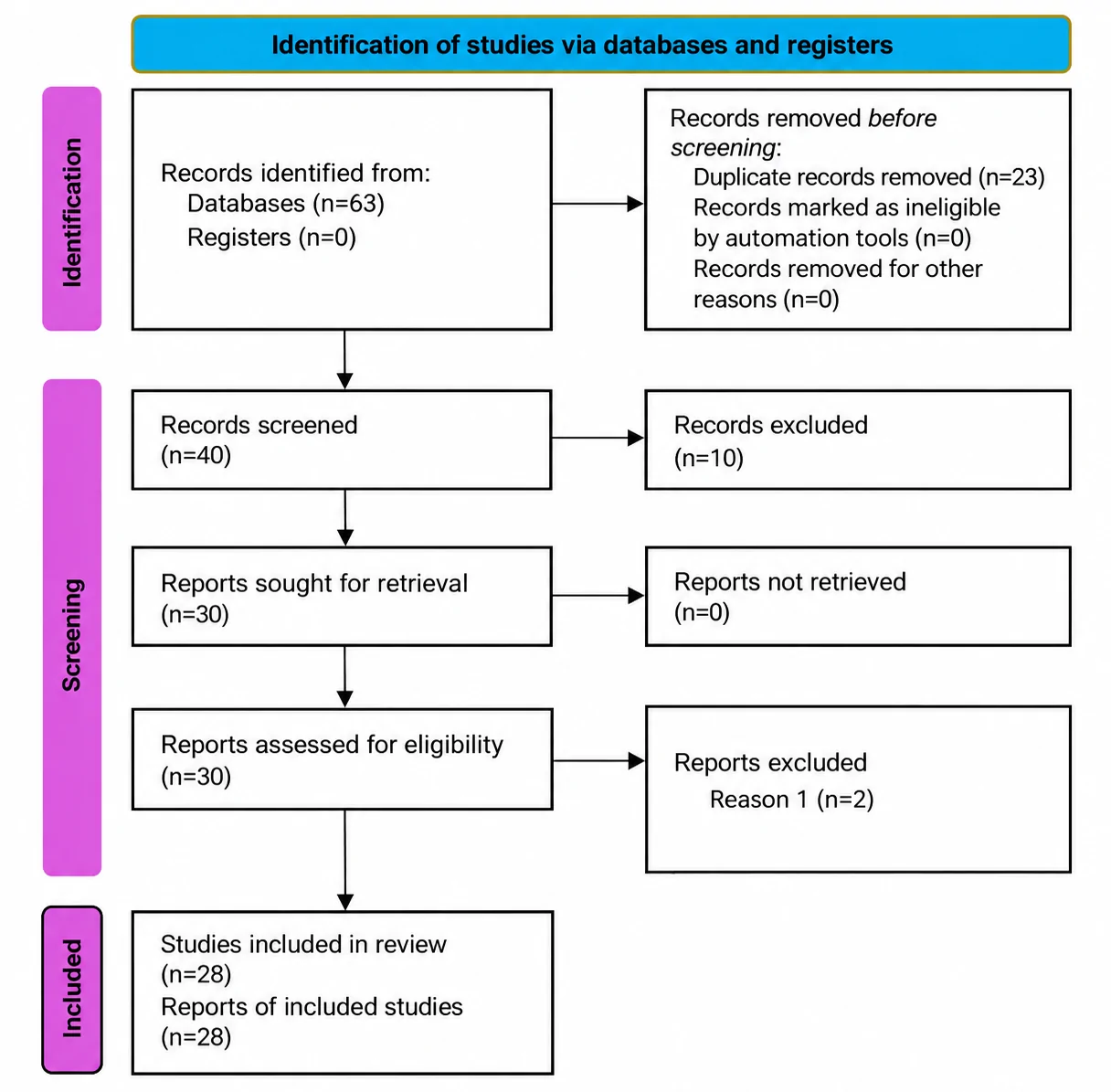
PRISMA-ScR (Preferred Reporting Items for Systematic Reviews and Meta-Analyses Extension for Scoping Reviews) flow diagram showing the study selection process.

### Characteristics of the Included Studies

The included studies were published between 2016 and 2025, with most publications concentrated from 2020 onward. In total, 7.1% (2/28) of the studies were published between 2016 and 2019, 46.4% (13/28) were published between 2020 and 2022, and 46.4% (13/28) were published between 2023 and 2025. Overall, 92.9% (26/28) of the studies were published between 2020 and 2025.

Regarding the objective of examining digital health and telehealth applications, 21.4% (6/28) of the studies evaluated interventions in which these technologies constituted a core component, while an additional 21.4% (6/28) incorporated them in a limited or secondary role. No such component was identified in 57.1% (16/28) of the studies. These applications included hospital-based patient engagement through bedside tablets [17], home-based screening supported by digital communication and image submission [18], and assessments of digital health system maturity [19]. To address the RQ on levels and patterns of integration between the frameworks, we examined the level of conceptual integration between the RE-AIM framework and theory of change or logic models across the included studies. Of the 28 included studies, strong integration was identified in 16 (57.1%), while moderate integration was observed in 10 (35.7%). Weak integration was identified in 3.6% (1/28) of the studies, and the classification was not applicable in 3.6% (1/28) of the studies.

Representative examples further illustrate these distinctions. Strong integration reflected an explicit connection between the causal or programmatic model and RE-AIM domains across intervention planning and evaluation, whereas moderate integration reflected complementary use of the 2 approaches without systematic articulation between causal assumptions and evaluative domains. In the study classified as weak integration, both approaches were identified, but no clear linkage between the proposed mechanisms and RE-AIM dimensions was described. Table 2 summarizes the publication period, presence of digital health or telehealth components, and level of integration between the RE-AIM framework and theory of change or logic models.

Representative studies further illustrate how these criteria were applied. Strong integration was observed when the causal or programmatic model was explicitly connected to RE-AIM domains across intervention planning and evaluation. This pattern was observed, for example, in the studies by Milgram et al [20], Williams et al [21], and Shuro et al [22]. Moderate integration reflected complementary but less systematically articulated use of the 2 approaches, as illustrated by Stadnick et al [23], Dolansky et al [24], Graco et al [25], and Ejskjaer et al [26]. Weak integration was identified only in the study by Duru et al [27], in which the logic model guided implementation and the RE-AIM framework guided evaluation without explicit mapping between the 2 approaches.

**Table 2.** Summary characteristics of the included studies (N=28).

Characteristics	Studies, n (%)
Year of publication	
2016-2019	2 (7.1)
2020-2022	13 (46.4)
2023-2025	13 (46.4)
Digital health or telehealth component	
Yes	6 (21.4)
Partial	6 (21.4)
No	16 (57.1)
Level of RE-AIM^a^–ToC^b^ integration	
Strong	16 (57.1)
Moderate	10 (35.7)
Weak	1 (3.6)
Not applicable	1 (3.6)

a RE-AIM: reach, effectiveness, adoption, implementation, and maintenance.

b ToC: theory of change.

### Functions of Theory of Change and Logic Models

To address the first RQ, we examined how the included studies applied the theory of change and logic models. Three primary functions were identified: program planning and design, implementation evaluation, and evaluation of mechanisms and impact. Studies applying these tools for program planning and design [28-31] used them to structure intervention stages, stakeholders, expected outcomes, and underlying assumptions. Studies focused on implementation evaluation [29,32-36] used logic models to structure implementation processes and support the interpretation of implementation outcomes, barriers, facilitators, and mechanisms.

A smaller subset of studies addressed the evaluation of mechanisms and impact [17,37,38]. Although less frequent, these studies demonstrated more advanced use of logic models to infer causal pathways.

## Discussion

### Principal Findings

The integration of the RE-AIM framework with theory of change or logic models aligns with broader developments in implementation science aimed at improving the design, evaluation, and sustainability of complex health interventions. Since its original formulation, the RE-AIM framework has been widely adopted as a pragmatic framework to enhance the public health impact of interventions by extending evaluation beyond efficacy to include reach, adoption, implementation, and maintenance [1,2]. In parallel, theory of change and logic models have been increasingly used in health and social programs to make explicit causal mechanisms, underlying assumptions, and contextual conditions through which interventions are expected to produce outcomes [3,4].

Within this broader context, the findings of the present scoping review indicate that empirical studies have increasingly combined these approaches to address known limitations of implementation evaluation, particularly when dealing with complex, adaptive, and context-sensitive interventions. By linking causal reasoning to structured implementation metrics, this integration may support greater transparency, interpretability, and learning across the intervention life cycle.

Despite the growing emphasis on digital health in policy and research agendas, digital health or telehealth constituted a core component of the intervention in 6 studies, while 6 additional studies incorporated such elements in a limited or secondary role. These included hospital-based patient engagement through bedside tablets [17], digital education and WhatsApp-based communication to support screening programs [18], and assessments of digital health system maturity [19].

Although digital health or telehealth components were identified in 12 of the 28 included studies, they constituted a core component in only 6, while the other 6 incorporated them in a limited or secondary role. This pattern suggests that the methodological gap relates less to the presence of these technologies than to the depth of their application within interventions integrating the RE-AIM framework with theory of change or logic models.

### Overview: Synthesis of Main Patterns

On the basis of cross-study analytic synthesis, four central patterns emerged across the 28 included studies. These patterns were derived from a comparative, cross-study synthesis of the extracted variables (including intervention context, telehealth components, integration level, and RE-AIM domain coverage) conducted iteratively by the research team through discussion and consensus. First, an increasingly systematic integration of the RE-AIM framework and theory of change was observed over time, with a marked intensification after 2020. Second, higher levels of methodological maturity were reported in interventions implemented in community settings and complex health systems, as illustrated by studies such as those by Glasgow et al [39] and Dougherty et al [32]. Third, the RE-AIM framework was most often applied in an instrumental manner, primarily to support monitoring and implementation-focused evaluation. Finally, persistent gaps were identified in long-term evaluation, particularly regarding the maintenance domain, as well as in the application of this integrated framework to digital health and telehealth interventions.

This scoping review identified 28 studies that explicitly integrated the RE-AIM framework with theory of change or logic models in the evaluation and implementation of health interventions, including digital health initiatives. The analysis demonstrated that the combined use of these frameworks has progressed in a heterogeneous yet consistent manner and has been applied to both structure intervention design and guide implementation and evaluation strategies. Overall, these findings support the value of integrating a well-established evaluative framework with an approach capable of explicating causal mechanisms and impact pathways, particularly in complex health system contexts.

A prominent pattern observed was the predominant use of logic models and theory of change as the conceptual foundation for formulating or adapting interventions, while the RE-AIM framework was primarily used as a framework for organizing implementation indicators and outcomes. Methodological and framework development studies, including those by Mash et al [40], Milgram et al [20], Pajer et al [29], and Glasgow et al [39], contributed to consolidating this articulation by demonstrating how clarification of mechanisms, assumptions, and contextual factors facilitates alignment between metrics and implementation processes.

Similarly, interventions in mental health [31,33], weight management programs [41], large-scale care coordination initiatives [36], caries prevention programs [30], and behavior change–oriented strategies [38,42,43] grounded their designs in explicit causal models, with evaluative operationalization conducted through the RE-AIM dimensions.

Six studies illustrated how integration between the frameworks supported implementation evaluation. This was evident in home-based colorectal cancer screening interventions using digital communication and image submission [18,44], community-based COVID-19 testing strategies [23], hospital-based patient engagement programs using tablets [17], the development of complex digital systems for maturity assessment [19], and large multinational initiatives focused on neonatal device quality and management [32]. In these contexts, theory of change enabled mapping of technological components, operational workflows, and impact assumptions, while the RE-AIM framework facilitated systematic assessment of reach, adoption, fidelity, implementability, and maintenance, which may strengthen coherence between intervention design and evaluation.

Notably, some interventions were represented by complementary protocol and implementation studies, allowing observation of how the RE-AIM framework and logic models evolved across different stages of the same program. For example, the CRC-SIM colorectal cancer screening initiative illustrated a progression from protocol design, where the implementation research logic model structured causal assumptions and evaluation planning, to empirical implementation, in which RE-AIM domains were operationalized through participation, completion, and feasibility outcomes [18,44]. This longitudinal articulation across study phases highlights how the combined use of causal models and implementation frameworks can support continuity between intervention design and real-world evaluation.

Support for learning cycles and intervention adaptation also emerged as a salient function of this integration. Studies by Sjoberg et al [36], Glasgow et al [39], Sivaramakrishnan et al [43], Fan et al [17], Gadsby et al [38], and Dougherty et al [32] showed that the simultaneous use of both frameworks enhanced interpretation of results and provided a structured basis for adjusting implementation strategies. These iterative processes were particularly relevant during the COVID-19 pandemic, when interventions required rapid modification in response to local conditions, resource availability, and changes in community behavior.

### Comparison With Prior Work

Previous studies have examined the RE-AIM framework and theory of change separately in health interventions. In a systematic review of 62 publications, Breuer et al [5] found that theory of change was used in intervention development and evaluation, although reports often provided limited detail on how it was developed and applied. Kwan et al [11], based on 17 clinical and community programs, reported the use of the RE-AIM framework in both planning and evaluation, with differences in the dimensions considered at each stage. In a subsequent systematic review of 157 articles, D’Lima et al [45] found that the RE-AIM framework was used predominantly for evaluation: 149 articles used the framework for evaluation, 3 used it for planning, and 5 used it for both purposes.

Our results provide a different perspective by focusing on studies in which the RE-AIM framework and theory of change or logic models were used together. Strong integration was identified in 16 of the 28 included studies, and moderate integration was identified in 10. One study showed weak integration, and the classification was not applicable in another. The variation across these categories indicates that the concurrent use of the approaches does not necessarily imply explicit conceptual or operational links between them. This distinction differs from the focus of previous studies that described how the RE-AIM framework [11,45] or theory of change [5] was applied, whereas our analysis examined the degree of integration when both approaches were present.

Evidence from digital interventions also helps contextualize our results. Jorge et al [46] examined 21 mobile health studies in a systematic review and meta-analysis based on the 5 RE-AIM dimensions. In our review, digital health or telehealth components were identified in 12 of the 28 studies. These technologies constituted a core component in 6 studies and had a limited or secondary role in another 6. This result indicates that, among studies combining the RE-AIM framework with theory of change or logic models, the gap lies less in the presence of digital technologies than in the depth of their application.

### Methodological Considerations

The assessment of integration between the RE-AIM framework and theory of change or logic models was based on the information reported in the included publications. Therefore, the classifications reflect the degree to which explicit links between the approaches could be identified in those publications. This consideration is particularly relevant because the included evidence comprised different types of publications, with varying levels of detail regarding the operationalization of the frameworks.

### Implications for Research and Practice

Despite growing global interest in the evaluation of digital health programs, only a small proportion of the included studies examined digital interventions in a robust manner. The studies by Fan et al [17], Liaw [19], Schliemann et al [18,44], and Dougherty et al [32] illustrate the potential of combining the RE-AIM framework and theory of change to capture key features of digital technologies, including rapid scalability, remote interactions, dependence on infrastructure, and the need for real-time monitoring. Nevertheless, the literature remains fragmented and insufficiently consolidated, underscoring the need for greater systematization in this emerging field.

Another persistent challenge concerns the maintenance dimension of the RE-AIM framework, which continues to be underexplored even in large-scale studies or those with substantial institutional investment, such as national health system initiatives or major hospital programs. Although some studies reported sustainability-related data [32,33,38,39], most did not provide sufficient medium- or long-term analyses to assess persistence, scale-up, or institutionalization of interventions.

Overall, the findings of this review underscore the relevance and potential of integrating the RE-AIM framework and theory of change to strengthen the evaluation and implementation of health interventions, including digital solutions. The articulation of causal mechanisms, implementation strategies, and evaluative domains was observed across clinical and community-based interventions, digital technologies, and organizational programs. At the same time, the review highlights the need for greater methodological rigor, deeper integration between frameworks, more transparent reporting of processes and mechanisms, and increased attention to the sustainability of interventions over time.

In practical terms, theory of change or logic models can be used during intervention planning to make assumptions, mechanisms, and expected pathways explicit, while the RE-AIM framework can guide the selection of indicators across reach, effectiveness, adoption, implementation, and maintenance. During implementation, these elements can be revisited together to assess whether observed outcomes are consistent with the proposed pathways and identify where adaptations may be required. Figure 2 illustrates these complementary roles across intervention planning, implementation, evaluation, and sustainability.

**Figure 2.**
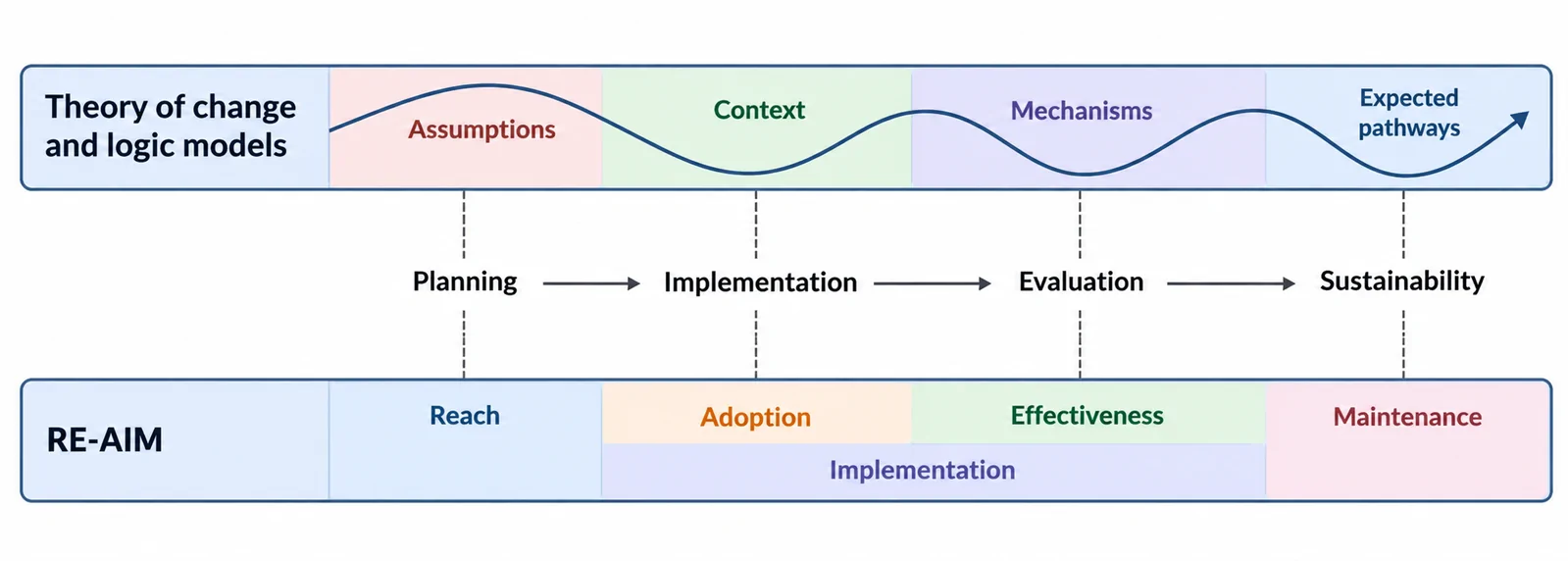
Complementary roles of theory of change or logic models and the reach, effectiveness, adoption, implementation, and maintenance (RE-AIM) framework across intervention stages. Theory of change and logic models articulate assumptions, context, mechanisms, and expected pathways across intervention planning, implementation, evaluation, and sustainability, while the RE-AIM framework provides complementary dimensions for assessing implementation and outcomes. The spatial positioning of the RE-AIM dimensions is illustrative and does not indicate an exclusive correspondence between individual RE-AIM dimensions and specific intervention stages.

### Limitations

This review has several limitations that should be considered when interpreting the findings. Although the search strategy was comprehensive and conducted across four international databases, terminology in implementation science varies substantially. Many studies use “logic models,” “program logic,” or “implementation research logic models” without explicitly labeling these approaches as “theory of change,” which may have influenced study identification and screening [3,4]. The methodological decision to treat theory of change and logic models as equivalent constructs was necessary to ensure search sensitivity but may have increased conceptual heterogeneity among the included studies.

Another limitation relates to the diversity of thematic areas and intervention types. The combined application of the RE-AIM framework and logic models was identified across multiple sectors, including primary care, oncology, immunization, neonatal devices, community programs, and digital health. This diversity limits the feasibility of quantitative synthesis or direct comparison across clinical areas. Heterogeneity in study designs, including realist evaluations, mixed methods studies, implementation pilots, protocols, and hybrid designs, further constrained the possibility of standardized synthesis. It also limited the specific focus on digital health and telehealth, which constituted a core component in only a subset of studies and played a limited or secondary role in others.

Finally, this review did not assess the methodological quality of the included studies in accordance with JBI recommendations for scoping reviews [13]. While appropriate for the objective of mapping the field, this decision means that the conclusions should be interpreted as exploratory and descriptive rather than as evidence of the effectiveness of specific approaches.

### Conclusions

This scoping review provides a structured mapping of how scientific literature has integrated the RE-AIM framework with theory of change or logic models in the implementation and evaluation of health interventions. The findings indicate that this integration has primarily been used as a methodological strategy to align causal reasoning with implementation-focused evaluation, supporting greater transparency and coherence in the assessment of complex interventions.

Overall, the review highlights the potential of combining the RE-AIM framework and theory of change to strengthen planning, implementation, and evaluation across diverse health contexts while also underscoring the need for more consistent operationalization, particularly regarding long-term sustainability and digital health applications. Future research should further advance this integrated approach to enhance learning, scalability, and sustained impact in real-world health systems.

For implementation scientists, digital health researchers, and health system evaluators, this combined approach may provide a practical basis for linking expected mechanisms of change with implementation outcomes, thereby informing the design and evaluation of future interventions.

## Supplementary material

10.2196/95809 Multimedia Appendix 1 Full search strategies used across databases.

10.2196/95809 Multimedia Appendix 2 Key terms and conceptual definitions.

10.2196/95809 Multimedia Appendix 3 Data extraction chart for the included studies (N=28).

10.2196/95809 Checklist 1 PRISMA-ScR checklist.

## Abbreviations

JBI: Joanna Briggs Institute
PRISMA-ScR: Preferred Reporting Items for Systematic Reviews and Meta-Analyses Extension for Scoping Reviews
RE-AIM: reach, effectiveness, adoption, implementation, and maintenance
RQ: research question
