# Supplementary material for: Integrating the RE-AIM Framework and Theory of Change in the Evaluation of Health Interventions: Scoping Review
Source: Online J Public Health Inform. 2026 Nov 11;18:e95809. doi: 10.2196/95809 (PMC13754177; doi:10.2196/95809)
Supplement: Supplementary file 1 [file ojphi-v18-e95809-s001.docx]

**Multimedia Appendix 2. Search strings by database.**

| **Database** | Search string (as executed) | Results |
| --- | --- | --- |
| **PubMed** | ("RE-AIM" OR REAIM) AND ("theory of change" OR "logic model" OR "program theory") AND (telehealth OR telemedicine OR eHealth OR health OR "digital health") | 22 |
| **Scopus** | TITLE-ABS-KEY("RE-AIM" OR REAIM) AND TITLE-ABS-KEY("theory of change" OR "logic model" OR "program theory") AND TITLE-ABS-KEY(telehealth OR telemedicine OR "tele-health" OR ehealth OR "e-health" OR mhealth OR "digital health") | 2 |
| **Web of Science – Core Collection** | TS=("theory of change") AND TS=("RE-AIM") | 7 |
| **Embase** | ('re-aim':ti,ab OR reaim:ti,ab) AND ('theory of change':ti,ab OR 'logic model':ti,ab OR 'program theory':ti,ab OR 'programme theory':ti,ab OR 'theories of change':ti,ab) AND (telehealth OR telemedicine OR ehealth OR health OR 'digital health') | 32 |
