# Supplementary material for: Integrating the RE-AIM Framework and Theory of Change in the Evaluation of Health Interventions: Scoping Review
Source: Online J Public Health Inform. 2026 Nov 11;18:e95809. doi: 10.2196/95809 (PMC13754177; doi:10.2196/95809)
Supplement: Supplementary file 2 [file ojphi-v18-e95809-s002.docx]

**Multimedia Appendix 3. Key terms and conceptual definitions.**

| **Term** | **Operational definition used in this review** |
| --- | --- |
| RE-AIM framework | An implementation science framework assessing interventions across five dimensions: Reach, Effectiveness, Adoption, Implementation, and Maintenance. In this review, RE-AIM was used as an evaluative structure to organize indicators related to implementation processes and outcomes. |
| Theory of Change / logic model | Theory of Change was considered the broader concept for articulating how and why an intervention is expected to produce change, including causal mechanisms, assumptions, contextual factors, and expected pathways. Logic models were considered structured representations of these pathways. |
| Levels of RE-AIM–ToC integration | Classified as strong when both frameworks jointly informed intervention design, implementation, and evaluation; moderate when Theory of Change or logic models informed planning and RE-AIM was primarily evaluative; and weak when both frameworks were present but not explicitly articulated. |
| Telehealth classification | Telehealth was coded as 'Yes' when digital or remote technologies constituted a core component of intervention delivery or evaluation; as 'Partial' when telehealth components were present but secondary or hybrid; and as 'No' when no telehealth component was reported. |
