# Supplementary material for: Integrating the RE-AIM Framework and Theory of Change in the Evaluation of Health Interventions: Scoping Review
Source: Online J Public Health Inform. 2026 Nov 11;18:e95809. doi: 10.2196/95809 (PMC13754177; doi:10.2196/95809)
Supplement: Supplementary file 3 [file ojphi-v18-e95809-s003.docx]

**Multimedia Appendix 4. Data extraction chart for the included studies (N = 28).**

| **No** | **Year** | **Authors** | | **Title** | **Journal** | | **Country/ Primary context** | | **Study design** | | **Type of intervention, program, or Service** | | **Digital health/telehealth component** | | **RE-AIM** | | **ToC/ Logic model** | | **RE-AIM–ToC Integration** | | **Framework role** | | **RE-AIM domains** | |  | | | | |  | | |  | | |  | | |  | |  | |  | |  |  |  |  |  |  |  |  |  |  |  |
| --- | --- | --- | --- | --- | --- | --- | --- | --- | --- | --- | --- | --- | --- | --- | --- | --- | --- | --- | --- | --- | --- | --- | --- | --- | --- | --- | --- | --- | --- | --- | --- | --- | --- | --- | --- | --- | --- | --- | --- | --- | --- | --- | --- | --- | --- | --- | --- | --- | --- | --- | --- | --- | --- | --- | --- |
| 1 | 2025 | | Mash et al. | Understanding implementation research | | African Journal of Primary Health Care & Family Medicine | | South Africa / primary care | | Methodological/conceptual article | | Not applicable (conceptual synthesis of implementation research) | | No | | Yes | | Yes | | Not applicable (methodological or conceptual article) | RE-AIM and implementation logic models were discussed conceptually as examples of implementation research frameworks, without empirical application. | | | RE-AIM domains were described conceptually as implementation outcomes but were not operationalized or empirically assessed. | |  |  |  | | |  | | |  | | |  |  |  |  |  |  |  |  |  |  |  |  |  |  |  |  |  |  |  |
| 2 | 2023 | | Milgram et al. | A Framework for Evaluating the Implementation of Biosimilar Drugs | | Canadian Journal of Hospital Pharmacy | | Canada / health care systems | | Framework development article | | Implementation of biosimilars in health systems | | No | | Yes | | Yes | | Strong | | A logic model describing biosimilar implementation activities and expected outcomes was developed to define the scope of the evaluation. Each component of the logic model was then examined across the five RE-AIM dimensions to generate evaluation questions and indicators, demonstrating explicit integration of the two approaches. | All five RE-AIM domains (Reach, Effectiveness, Adoption, Implementation, and Maintenance) were explicitly applied to the activities and expected outcomes identified in the logic model to develop the evaluation framework. | | |  | | |  | | |  | | |  | | |  | |  | |  | |  |  |  |  |  |  |  |  |  |  |  |  |
| 3 | 2024 | | Pajer et al. | Participatory logic model for a precision child and youth mental health start-up | | Frontiers in Health Services | | Canada / child and youth mental health | | Scoping review + participatory case study | | Precision mental health program for children and youth | | Partial | | Yes | | Yes | | Strong | Participatory logic model co-created with stakeholders to guide program planning, implementation, and evaluation; RE-AIM explicitly informed the development of logic model components and their measurement. | | | All five RE-AIM domains were explicitly used to inform logic model development: Reach informed participants and stakeholders; Effectiveness informed outcomes and measurement; Adoption informed participation and outputs; Implementation informed tracking of outputs and outcomes; and Maintenance informed long-term outcomes and impact. | |  |  |  | | |  | | |  | | |  |  |  |  |  |  |  |  |  |  |  |  |  |  |  |  |  |  |  |
| 4 | 2025 | | Glasgow et al. | Use of implementation logic models in the Quadruple Aim QUERI | | Implementation Science Communications | | United States / Veterans Health Administration | | Series of implementation studies (program QUERI) | | Multiple interventions for care coordination and quality improvement | | Partial | | Yes | | Yes | | Strong | Implementation logic models used to articulate strategies, contextual determinants, and outcomes; RE-AIM applied to guide planning, evaluation, and interpretation of implementation outcomes across QUERI initiatives. | | | Reach, Adoption, Implementation, and Maintenance explicitly addressed across implementation projects, with Effectiveness considered in relation to Quadruple Aim outcomes; domains operationalized within real-world implementation studies rather than only conceptually. | |  |  |  | | |  | | |  | | |  |  |  |  |  |  |  |  |  |  |  |  |  |  |  |  |  |  |  |
| 5 | 2023 | | Stadnick et al. | Community-engaged optimization of COVID-19 rapid evaluation and testing experiences | | Roll-out implementation optimization (ROIO) trial / study protocol | | United States / community clinics (FQHC) | | Implementation trial (ROIO) / protocol | | Community-based COVID-19 testing strategies | | No | | Yes | | Yes | | Moderate | Co-designed Theory of Change used to guide intervention design and community engagement strategies; PRISM/RE-AIM used to structure implementation optimization and evaluation processes. | | | All five RE-AIM domains were prospectively operationalized for assessment during the ROIO trial. | |  |  |  | | |  | | |  | | |  |  |  |  |  |  |  |  |  |  |  |  |  |  |  |  |  |  |  |
| 6 | 2021 | | Fiechtner et al. | Design and Approach of the Healthy Weight Clinic Pediatric Weight Management Intervention Package and Implementation | | Childhood Obesity | | United States / pediatric primary care | | Effectiveness-implementation hybrid (type III) / protocol | | Pediatric weight management program in pediatric clinic (Healthy Weight Clinic PWMI) | | Partial | | Yes | | Yes | | Moderate | Implementation Research Logic Model used to define facilitators, barriers, strategies, and mechanisms; RE-AIM used to structure evaluation of implementation outcomes within a hybrid effectiveness-implementation design. | | | RE-AIM was planned to evaluate pilot outcomes, including implementation outcomes and effectiveness; specific domain-level measures were not fully detailed in the article. | |  |  |  | | |  | | |  | | |  |  |  |  |  |  |  |  |  |  |  |  |  |  |  |  |  |  |  |
| 7 | 2022 | | Sjoberg et al. | Adaptations to relational facilitation for two national care coordination programs during COVID-19 | | Frontiers in Health Services | | United States / national care coordination programs (VA) | | Implementation case study | | Relational facilitation strategy for care coordination | | Partial | | Yes | | Yes | | Strong | Logic model used to track and structure adaptations to the relational facilitation implementation strategy; RE-AIM dimensions used to classify and analyze adaptations across implementation outcomes. | | | The broader QA QUERI evaluation addressed all five RE-AIM domains; in the adaptation analysis reported in this study, adaptations were classified under Adoption (n=6) and Implementation (n=2). | |  |  |  | | |  | | |  | | |  |  |  |  |  |  |  |  |  |  |  |  |  |  |  |  |  |  |  |
| 8 | 2023 | | Schliemann et al. | Implementation of a home-based colorectal cancer screening intervention in Malaysia (CRC-SIM) | | BMC Cancer | | Malaysia / colorectal cancer screening | | Quasi-experimental implementation study | | Home-based iFOBT screening with mobile phone and WhatsApp support | | Yes | | Yes | | Yes | | Strong | Implementation Research Logic Model used to guide intervention design and implementation planning; RE-AIM framework used to evaluate implementation outcomes, complemented by measures of acceptability, appropriateness, and feasibility. | | | Reach, Effectiveness, Adoption, and Implementation were explicitly assessed; Reach through study participation, Effectiveness and Adoption through iFOBT completion and follow-up outcomes, and Implementation through intervention delivery, timelines, challenges, and adaptations. Maintenance was not clearly evaluated within the study period. | |  |  |  | | |  | | |  | | |  |  |  |  |  |  |  |  |  |  |  |  |  |  |  |  |  |  |  |
| 9 | 2022 | | Wang L. et al. | Evaluating an oral health programme for the prevention of dental decay among school-aged children in China | | BMJ Open | | China / school oral health | | Mixed-methods study protocol with economic evaluation | | CIODC dental caries prevention program among schoolchildren | | No | | Yes | | Yes | | Strong | Logic model used to structure the process evaluation, link programme inputs, activities, and outcomes, and guide qualitative data collection and analysis; RE-AIM framework used to structure the process evaluation across its five domains. | | | All five RE-AIM domains were explicitly operationalized in the evaluation protocol, with predefined data sources, indicators, and outputs for Reach, Effectiveness, Adoption, Implementation, and Maintenance; the article reports the evaluation protocol rather than the final empirical findings. | |  |  |  | | |  | | |  | | |  |  |  |  |  |  |  |  |  |  |  |  |  |  |  |  |  |  |  |
| 10 | 2024 | | Haun et al. | A Partner-Engaged Approach to Developing an Implementation Research Logic Model for a Traumatic Brain Injury-Intensive Evaluation and Treatment Program | | Journal of Head Trauma Rehabilitation | | United States / TBI rehabilitation in veterans | | Participatory-based concurrent mixed-methods quality improvement study / IRLM development | | Intensive evaluation and treatment program for TBI (IETP) | | No | | Yes | | Yes | | Strong | Implementation Research Logic Model developed through partner engagement to integrate program characteristics, CFIR-informed determinants, implementation strategies, mechanisms, and outcomes; RE-AIM, combined with the Implementation Outcomes Framework, was used to define and organize implementation, service, and patient-level outcomes within the IRLM. | | | All five RE-AIM domains were explicitly operationalized within the IRLM: Reach through referrals and enrollment; Effectiveness through patient outcomes up to 6-month follow-up; Adoption through staff buy-in, module use, and site expansion; Implementation through acceptability, feasibility, and program fidelity; and Maintenance through program retention and cost. | |  |  |  | | |  | | |  | | |  |  |  |  |  |  |  |  |  |  |  |  |  |  |  |  |  |  |  |
| 11 | 2023 | | Sivaramakrishnan et al. | A mixed method evaluation of a theory based intervention to reduce sedentary behaviour in contact centres | | PLOS ONE | | United Kingdom / contact centers | | Stepped-wedge cluster randomized feasibility trial / process evaluation | | Stand Up for Health intervention to reduce workplace sedentary time | | No | | Yes | | Yes | | Strong | Theory of Change guided intervention development, adaptation, and centre-specific action plans; RE-AIM structured the process evaluation of reach, effectiveness, adoption, implementation, and maintenance, with Implementation explicitly assessing fidelity to programme theory rather than to specific intervention activities. | | | All five RE-AIM domains were empirically examined: Reach through programme availability, acceptance, appeal, participation, and barriers/enablers; Effectiveness through perceived benefits and quantitative outcomes; Adoption through the proportion of targeted centres participating; Implementation through programme theory elements and unintended consequences; and Maintenance through centres’ plans to continue the SUH programme. | |  |  |  | | |  | | |  | | |  |  |  |  |  |  |  |  |  |  |  |  |  |  |  |  |  |  |  |
| 12 | 2021 | | Dolansky et al. | Pre-Implementation of the Age-Friendly Health Systems Evidence-Based 4Ms Framework in a Multi-State Convenient Care Practice | | Worldviews on Evidence-Based Nursing | | United States / MinuteClinics (CVS Health) | | Quality improvement study / pre-implementation | | Integration of the 4Ms Framework (Age-Friendly Health Systems) | | No | | Yes | | Yes | | Moderate | RE-AIM used to guide data collection and pre-implementation planning and to inform meaningful components of the logic model developed for implementation of the 4Ms Framework. | | | RE-AIM domains informed data collection and development of the logic model during the pre-implementation phase; specific operationalization of individual RE-AIM domains cannot be determined from the available abstract. | |  |  |  | | |  | | |  | | |  |  |  |  |  |  |  |  |  |  |  |  |  |  |  |  |  |  |  |
| 13 | 2024 | | Fan et al. | Experience of a tertiary acute care hospital in Southeast Asia in initiating patient engagement with the aid of digital solutions | | Frontiers in Health Services | | Singapore / tertiary hospital | | Mixed-methods implementation study | | Implementation of bedside tablets for patient engagement | | Yes | | Yes | | Yes | | Strong | Implementation Research Logic Model used to describe and structure hospital-wide implementation processes; RE-AIM applied to evaluate preliminary implementation outcomes and adoption of bedside tablets. | | | All five RE-AIM domains were explicitly assessed: Reach through coverage of eligible general-ward patients; Effectiveness through qualitative patient feedback; Adoption through staff and patient uptake; Implementation through delivery as intended; and Maintenance through continued monitoring of monthly adoption rates. | |  |  |  | | |  | | |  | | |  |  |  |  |  |  |  |  |  |  |  |  |  |  |  |  |  |  |  |
| 14 | 2024 | | Shuro et al. | Enhancing childhood immunization coverage in Mozambique and Malawi: 'Let's talk about vaccines' | | PLOS ONE | | Mozambique and Malawi / childhood immunization | | Mixed-methods prospective quasi-experimental and process evaluation protocol | | Community-based participatory project + HCD for vaccination of children under 2 years | | No | | Yes | | Yes | | Strong | Theory of Change used to articulate pathways from implementation strategies to expected outcomes and refined throughout the evaluation; RE-AIM used to guide planning, evaluation, and analysis of effectiveness and implementation outcomes. | | | All five RE-AIM domains were prospectively incorporated into the evaluation: Reach, Effectiveness, Adoption, Implementation, and Maintenance. Maintenance was planned but acknowledged as potentially only partially assessable within the project timeframe. | |  |  |  | | |  | | |  | | |  |  |  |  |  |  |  |  |  |  |  |  |  |  |  |  |  |  |  |
| 15 | 2022 | | Weber et al. | A clinical-academic partnership to develop a family management intervention for parents of preterm infants | | Journal of Clinical Nursing | | United States / neonatal ICU | | Longitudinal qualitative descriptive study | | PREEMIE PROGRESS family management intervention in the neonatal ICU | | No | | Yes | | Yes | | Strong | Partnership Logic Model used deductively to examine how the clinical-academic partnership contributed to intervention development; RE-AIM used to structure a priori coding of stakeholder feedback and identify opportunities to improve intervention reach, effectiveness, adoption, implementation, and maintenance. | | | All five RE-AIM dimensions were incorporated as a priori codes in the qualitative analysis of stakeholder feedback during intervention development and refinement; they informed intervention improvement rather than a formal evaluation of implementation or effectiveness outcomes. | |  |  |  | | |  | | |  | | |  |  |  |  |  |  |  |  |  |  |  |  |  |  |  |  |  |  |  |
| 16 | 2022 | | Schliemann et al. | Implementation of a colorectal cancer screening intervention in Malaysia (CRC-SIM) in the context of a pandemic: study protocol | | BMJ Open | | Malaysia / colorectal cancer screening | | Study protocol for a quasi-experimental mixed-methods implementation study | | CRC-SIM home-based screening intervention with digital media support | | Yes | | Yes | | Yes | | Strong | Implementation Research Logic Model used to map determinants, implementation strategies, mechanisms, and outcomes and to inform intervention implementation; RE-AIM used to define implementation evaluation outcomes and measures within the mixed-methods quasi-experimental study. | | | All five RE-AIM dimensions were operationalized through predefined quantitative and qualitative outcome measures: Reach through participation and intervention exposure; Effectiveness through service- and patient-related outcomes; Adoption through staff training and uptake across settings; Implementation through fidelity, adherence, resource use, and costs; and Maintenance through willingness to sustain the intervention and attrition. Measures were planned but not yet empirically assessed because this was a study protocol. | |  |  |  | | |  | | |  | | |  |  |  |  |  |  |  |  |  |  |  |  |  |  |  |  |  |  |  |
| 17 | 2020 | | Gadsby EW et al. | Impact of a community-based pilot intervention to tackle childhood obesity: a 'whole-system approach' case study | | BMC Public Health | | United Kingdom / bairro urbano of Londres | | Mixed-methods case study | | Go-Golborne community childhood obesity prevention program | | No | | Yes | | Yes | | Stromg | Theory of Change and logic model used to articulate programme assumptions, activities, outputs, and expected outcomes and to guide qualitative analysis; RE-AIM used to guide data collection, management, analysis, and synthesis of findings across public health impact dimensions. | | | All five RE-AIM dimensions were empirically assessed: Reach through participation of children, families, schools, and community partners; Effectiveness/Efficacy through changes in knowledge, attitudes, behaviours, environments, and weight outcomes; Adoption through organisational and stakeholder participation; Implementation through programme delivery, fidelity, adaptation, training, resources, and contextual influences; and Maintenance through sustained partnerships and integration of programme actions, resources, and practices into local organisations. | |  |  |  | | |  | | |  | | |  |  |  |  |  |  |  |  |  |  |  |  |  |  |  |  |  |  |  |
| 18 | 2019 | | Williams et al. | Evaluation of a Physical health plan for people with psychosis: a protocol for a quality improvement study | | Pilot and Feasibility Studies | | United Kingdom / community mental health services | | Protocol for an exploratory pilot quality improvement study | | Physical health plan (PHP) for people with psychosis | | Yes | | Yes | | Yes | | Strong | Theory of Change used to articulate, test, and refine assumptions about how the PHP is expected to work and to guide the exploratory study; RE-AIM used to structure the implementation evaluation, including predefined implementation outcomes within the pilot. | | | RE-AIM was planned to guide the implementation evaluation. Reach, Efficacy, and Maintenance were explicitly operationalized through predefined pilot outcomes; Adoption and Implementation informed the broader assessment of organisational uptake, implementation processes, training, adaptations, and implementation challenges. No empirical RE-AIM results were reported because this was a study protocol. | |  |  |  | | |  | | |  | | |  |  |  |  |  |  |  |  |  |  |  |  |  |  |  |  |  |  |  |
| 19 | 2020 | | Abhyankar et al. | Implementing pelvic floor muscle training for women with pelvic organ prolapse: a realist evaluation of different delivery models | | BMC Health Services Research | | United Kingdom / NHS services | | Longitudinal qualitative multiple-case study / realist evaluation | | Pelvic floor muscle training (PFMT) delivery models | | No | | Yes | | Yes | | Strong | Theory of Change developed, tested, and refined through realist CMO configurations to explain how, why, for whom, and under what contextual conditions PFMT implementation worked; RE-AIM used to assess translational and implementation outcomes, with observed outcomes explicitly mapped onto RE-AIM dimensions. | | | Reach, Effectiveness, Adoption, and Implementation were empirically assessed and integrated with the realist CMO analysis. Maintenance was defined within the RE-AIM framework but could not be assessed because study sites were not followed beyond the project period. | |  |  |  | | |  | | |  | | |  |  |  |  |  |  |  |  |  |  |  |  |  |  |  |  |  |  |  |
| 20 | 2025 | | Graco et al. | Polysomnographic titration of non-invasive ventilation in motor neurone disease (3TLA): protocol for a process evaluation of a clinical trial | | Trials | | Australia / rehabilitation and sleep centers | | Mixed-methods process evaluation protocol alongside a multicentre RCT | | PSG-assisted initiation and titration of NIV in people with MND | | No | | Yes | | Yes | | Moderate | Logic model used to define the intervention, mechanisms of impact, contextual factors, assumptions, and anticipated outcomes; RE-AIM used to structure implementation outcomes and quantitative process evaluation across all five dimensions, alongside TDF for barriers and enablers and TFA for acceptability. | | | All five RE-AIM dimensions were explicitly operationalized in the process evaluation protocol. Reach assesses participation and representativeness; Effectiveness includes behavioural, clinical, service-use, and participant outcomes; Adoption addresses acceptability, feasibility, uptake, barriers, and enablers; Implementation focuses on fidelity and intervention delivery; and Maintenance assesses the potential integration of the intervention into usual practice and health professionals’ views about its future use. | |  |  |  | | |  | | |  | | |  |  |  |  |  |  |  |  |  |  |  |  |  |  |  |  |  |  |  |
| 21 | 2025 | | Dougherty et al. | From warehouse to ward: applying implementation research methods to the device identification, qualification, distribution, and management process within the NEST360 alliance | | BMC Global Public Health | | Kenya, Malawi, Nigeria, Tanzania / neonatal hospitals | | Retrospective mixed-methods implementation study | | Processes for qualification, distribution, and management of newborn devices (NEST360) | | No | | Yes | | Yes | | Strong | Implementation Research Logic Model developed to map contextual factors, implementation strategies, mechanisms, and implementation outcomes; RE-AIM used to define and assess implementation outcomes within the IRLM. | | | All five RE-AIM domains were empirically operationalized: Reach, Effectiveness, Adoption, Implementation, and Maintenance were assessed using quantitative programmatic data and qualitative findings across facility, country, and alliance levels. | |  |  |  | | |  | | |  | | |  |  |  |  |  |  |  |  |  |  |  |  |  |  |  |  |  |  |  |
| 22 | 2025 | | Liaw et al. | Methodologies for quality measurement, monitoring, and evaluation | | Digital Health Maturity Quality Interoperability and Innovation (book chapter) | | Internacional / digital health systems | | Theoretical-methodological chapter | | Digital health maturity assessment (DHM3, QIMME) | | Yes | | Yes | | Yes | | Moderate | Logic models used to structure digital health maturity pathways and program indicators; RE-AIM-informed evaluation approaches discussed conceptually to support monitoring, quality improvement, and organizational learning. | | | RE-AIM domains discussed conceptually in relation to digital health maturity assessment and evaluation processes, without empirical operationalization. | |  |  |  | | |  | | |  | | |  |  |  |  |  |  |  |  |  |  |  |  |  |  |  |  |  |  |  |
| 23 | 2022 | | Chan & Glass | Delivering a holistic hospital-to-home framework to support family caregivers of persons with dementia: Protocol for a feasibility study | | Journal of Advanced Nursing | | Singapore / tertiary hospital and community | | Multimethod feasibility study / protocol | | Carer Matters hospital-to-community support program for caregivers of people with dementia | | Partial | | Yes | | Yes | | Moderate | Theory of Change used to guide program design and feasibility evaluation; RE-AIM used as a framework to synthesize implementation and outcome findings. | | | Reach, Effectiveness, Adoption, Implementation, and Maintenance planned as evaluation domains through RE-AIM synthesis, but not yet empirically assessed at the protocol stage. | |  |  |  | | |  | | |  | | |  |  |  |  |  |  |  |  |  |  |  |  |  |  |  |  |  |  |  |
| 24 | 2022 | | Duru et al. | Implementing shared decision making (SDM) for diabetes prevention: preliminary results and lessons learned | | Journal of General Internal Medicine (supplement) | | United States / primary care (UCLA) | | Program report / implementation study (conference abstract) | | Shared decision-making program with pharmacists for type 2 diabetes prevention | | Partial | | Yes | | Yes | | Weak | RE-AIM and the logic model were used for distinct but complementary purposes: the logic model guided the implementation phase through five core functions, whereas RE-AIM guided the evaluation of the intervention. No explicit mapping or systematic linkage between the logic model and RE-AIM domains was described in the conference abstract. | | | RE-AIM guided evaluation through metrics related to reach and representativeness of enrollment, 12-month weight change, PCP perceptions of program value, and participant assessment of shared decision-making quality. | |  |  |  | | |  | | |  | | |  |  |  |  |  |  |  |  |  |  |  |  |  |  |  |  |  |  |  |
| 25 | 2022 | | Ejskjaer et al. | Interim Real-World Ten-Site Data from the National Danish PRO Diabetes Multisector Study (M-PRODIA) | | Diabetes (supplemento) | | Denmark / multiple diabetes services | | Real-world multicenter implementation study / interim conference abstract | | PRO-based care model for diabetes (VBHC-PRO-DIA) | | Yes | | Yes | | Yes | | Moderate | A PRO diabetes logic model was developed to describe how PRO could drive health value, while RE-AIM was used to assess the public health potential of the multicenter real-world PRO pilot study. | | | RE-AIM was used to evaluate the public health potential of the real-world multicenter PRO pilot; interim results reported acceptability and perceived benefits among >460 people with diabetes and 30 healthcare professionals, without domain-specific results for all five RE-AIM dimensions. | |  |  |  | | |  | | |  | | |  |  |  |  |  |  |  |  |  |  |  |  |  |  |  |  |  |  |  |
| 26 | 2020 | | Hiatt et al. | The San Francisco Cancer Initiative: Addressing disparities in colorectal cancer screening in community health centers | | Cancer Epidemiology, Biomarkers & Prevention (supplemento) | | United States / community health centers in San Francisco | | Program evaluation | | SF CAN initiative to increase FIT screening in vulnerable populations | | No | | Yes | | Yes | | Moderate | program logic used to structure the SF CAN initiative and guide implementation planning; RE-AIM used to support monitoring and evaluation of screening uptake and implementation outcomes. | | | Reach and Adoption reflected through screening uptake; Implementation addressed via program delivery processes; Effectiveness and Maintenance implied but not fully operationalized. | |  |  |  | | |  | | |  | | |  |  |  |  |  |  |  |  |  |  |  |  |  |  |  |  |  |  |  |
| 27 | 2020 | | Rocha et al. | Using implementation science to examine impact of a social responsibility agenda on addressing cancer health disparities in Ceara, Brazil | | Journal of Clinical Oncology (congress abstract) | | Brazil / cancer center in Ceara | | Study proposal / implementation framework | | Agenda of Responsabilidade Social for reduzir disparidades in câncer | | No | | Yes | | Yes | | Moderate | Logic model developed to structure the social responsibility agenda and define implementation pathways; RE-AIM used as an evaluation framework to guide assessment of cancer care disparities and implementation outcomes. | | | Reach, Adoption, and Implementation were planned as evaluation domains within the proposed implementation framework; Effectiveness and Maintenance were conceptually addressed but not empirically assessed at the proposal stage. | |  |  |  | | |  | | |  | | |  |  |  |  |  |  |  |  |  |  |  |  |  |  |  |  |  |  |  |
| 28 | 2016 | | Gallegos et al. | Living well multicultural – does it change behaviours in migrants to reduce the risk of chronic disease? | | Revista Española de Nutrición Humana y Dietética (supplemento) | | Australia / migrant and refugee communities | | Pre-post program evaluation | | Living Well Multicultural lifestyle modification program | | No | | Yes | | Yes | | Moderate | Logic model used to structure the culturally adapted lifestyle intervention and expected behaviour change pathways; RE-AIM applied to evaluate program reach, adherence, and behavioural outcomes. | | | Reach and Adoption reflected through participation of migrant communities; Effectiveness assessed through behavioural and anthropometric outcomes; Implementation described through program delivery processes; Maintenance suggested conceptually but not fully evaluated in the abstract. | |  |  |  | | |  | | |  | | |  |  |  |  |  |  |  |  |  |  |  |  |  |  |  |  |  |  |  |

**References of the included studies**

1. [Reference 40] Mash R, Nyasulu J, Malan Z, Hirschhorn L. Understanding implementation research. Afr J Prim Health Care Fam Med. Jun 3, 2025;17(2):e1-e7. [doi: 10.4102/phcfm.v17i2.4934] [Medline: 40459100]
2. [Reference 20] Milgram L, Wheeler S, Adamic A, Loncar M, Guirguis M, McCabe BJ. A framework for evaluating the implementation of biosimilar drugs. Can J Hosp Pharm. 2023;76(2):109-116. [doi: 10.4212/cjhp.3272] [Medline: 36998755]
3. [Reference 29] Pajer K, Honeywell C, Howley H, et al. Participatory logic model for a precision child and youth mental health start-up: scoping review, case study, and lessons learned. Front Health Serv. 2024;4:1405426. [doi: 10.3389/frhs.2024.1405426] [Medline: 39483443]
4. [Reference 39] Glasgow RE, McCreight MS, Morgan B, et al. Use of implementation logic models in the Quadruple Aim QUERI: conceptualization and evolution. Implement Sci Commun. Jan 16, 2025;6(1):10. [doi: 10.1186/s43058-024-00678-6] [Medline: 39819373]
5. [Reference 23] Stadnick NA, Laurent LC, Cain KL, et al. Community-engaged optimization of COVID-19 rapid evaluation and testing experiences: roll-out implementation optimization trial. Implement Sci. Oct 2, 2023;18(1):46. [doi: 10.1186/s13012-023-01306-y] [Medline: 37784200]
6. [Reference 41] Fiechtner L, Castro I, Ayala SG, et al. Design and approach of the healthy weight clinic pediatric weight management intervention package and implementation: Massachusetts-CORD 3.0. Child Obes. Sep 2021;17(S1):S48-S54. [doi: 10.1089/chi.2021.0177] [Medline: 34569841]
7. [Reference 36] Sjoberg H, Kenney RR, Morgan B, et al. Adaptations to relational facilitation for two national care coordination programs during COVID-19. Front Health Serv. 2022;2:952272. [doi: 10.3389/frhs.2022.952272] [Medline: 36925807]
8. [Reference 18] Schliemann D, Ramanathan K, Ibrahim Tamin NS, et al. Implementation of a home-based colorectal cancer screening intervention in Malaysia (CRC-SIM). BMC Cancer. Jan 6, 2023;23(1):22. [doi: 10.1186/s12885-022-10487-6] [Medline: 36609260]
9. [Reference 30] Wang L, Liu H, Wang L, et al. Evaluating an oral health programme for the prevention of dental decay among school-aged children in China: protocol for a mixed-methods study based on the RE-AIM framework. BMJ Open. Sep 2022;12(9):e061601. [doi: 10.1136/bmjopen-2022-061601] [Medline: 36180124]
10. [Reference 33] Haun JN, Melillo C, Schneider T, et al. A partner-engaged approach to developing an implementation research logic model for a traumatic brain injury-intensive evaluation and treatment program. J Head Trauma Rehabil. 2024;39(6):435-445. [doi: 10.1097/HTR.0000000000000988] [Medline: 39038102]
11. [Reference 43] Sivaramakrishnan D, Baker G, Parker RA, Manner J, Lloyd S, Jepson R. A mixed method evaluation of a theory based intervention to reduce sedentary behaviour in contact centres- the stand up for health stepped wedge feasibility study. PLoS One. 2023;18(12):e0293602. [doi: 10.1371/journal.pone.0293602] [Medline: 38100490]
12. [Reference 24] Dolansky MA, Pohnert A, Ball S, McCormack M, Hughes R, Pino L. Pre-implementation of the age-friendly health systems evidence-based 4Ms framework in a multi-state convenient care practice. Worldviews Evid Based Nurs. Apr 2021;18(2):118-128. [doi: 10.1111/wvn.12498] [Medline: 33856117]
13. [Reference 17] Fan PE, Lim SH, Sim GH, et al. Experience of a tertiary acute care hospital in Southeast Asia in initiating patient engagement with the aid of digital solutions. Front Health Serv. 2024;4:1416386. [doi: 10.3389/frhs.2024.1416386] [Medline: 39411515]
14. [Reference 22] Shuro L, Lawrence E, Knight L, Schneider H, Tabana H. Enhancing childhood immunization coverage in Mozambique and Malawi: study protocol of a mixed methods evaluation of the “let’s talk about vaccines” multisite community-based participatory project. PLoS One. 2024;19(11):e0311052. [doi: 10.1371/journal.pone.0311052] [Medline: 39565783]
15. [Reference 31] Weber AM, Voos KC, Bakas TM, et al. A clinical-academic partnership to develop a family management intervention for parents of preterm infants. J Clin Nurs. Feb 2022;31(3-4):390-405. [doi: 10.1111/jocn.15929] [Medline: 34219302]
16. [Reference 44] Schliemann D, Ramanathan K, Ibrahim Tamin NS, et al. Implementation of a colorectal cancer screening intervention in Malaysia (CRC-SIM) in the context of a pandemic: study protocol. BMJ Open. Sep 1, 2022;12(9):e058420. [doi: 10.1136/bmjopen-2021-058420] [Medline: 36581978]
17. [Reference 38] Gadsby EW, Hotham S, Eida T, Lawrence C, Merritt R. Impact of a community-based pilot intervention to tackle childhood obesity: a “whole-system approach” case study. BMC Public Health. Nov 30, 2020;20(1):1818. [doi: 10.1186/s12889-020-09694-2] [Medline: 33256660]
18. [Reference 21] Williams J, Sevdalis N, Gaughran F. Evaluation of a physical health plan for people with psychosis: a protocol for a quality improvement study. Pilot Feasibility Stud. 2019;5:8. [doi: 10.1186/s40814-019-0396-7] [Medline: 30675375]
19. [Reference 37] Abhyankar P, Wilkinson J, Berry K, et al. Implementing pelvic floor muscle training for women with pelvic organ prolapse: a realist evaluation of different delivery models. BMC Health Serv Res. Oct 1, 2020;20(1):910. [doi: 10.1186/s12913-020-05748-8] [Medline: 32998714]
20. [Reference 25] Graco M, Berlowitz DJ, Sawyer A, et al. Polysomnographic titration of non-invasive ventilation in motor neurone disease (3TLA): protocol for a process evaluation of a clinical trial. Trials. Mar 6, 2025;26(1):79. [doi: 10.1186/s13063-025-08784-z] [Medline: 40050976]
21. [Reference 32] Dougherty K, Kirby RP, Claud K, et al. From warehouse to ward: applying implementation research methods to the device identification, qualification, distribution, and management process within the Newborn Essential Solutions and Technologies (NEST360) alliance. BMC Glob Public Health. Aug 2, 2025;3(1):67. [doi: 10.1186/s44263-025-00185-3] [Medline: 40750904]
22. [Reference 19] Liaw ST. Methodologies for quality measurement, monitoring, and evaluation. In: Liaw ST, Jonnagaddala J, Godinho MA, Wilkins-Wong ZS, editors. Digital Health Maturity: Quality, Interoperability, and Innovation. Academic Press; 2026:157-177. [doi: 10.1016/B978-0-323-95260-6.00006-X]
23. [Reference 28] Chan EY, Glass GF Jr. Delivering a holistic hospital-to-home framework to support family caregivers of persons with dementia: protocol for a feasibility study. J Adv Nurs. May 2022;78(5):1513-1523. [doi: 10.1111/jan.15210] [Medline: 35285537]
24. [Reference 27] Duru OK, Maranon R, Moin T, et al. Implementing shared decision making (SDM) for diabetes prevention: preliminary results and lessons learned. J Gen Intern Med. 2022;37(Suppl 2):S573-S574.
25. [Reference 26] Ejskjaer N, Kjaer PH, Berthelsen DB, et al. 962-P: Interim real-world ten-site data from the National Danish PRO Diabetes Multisector Study (M-PRODIA) engaging 460 persons with diabetes and 30 health care professionals shows high acceptability and benefits. Diabetes. 2022;71(Supplement_1). [doi: 10.2337/db22-962-P]
26. [Reference 34] Hiatt R, Rachocki C, Potter MB, Somsouk M, Ofman D. Abstract C123: the San Francisco Cancer Initiative: addressing disparities in colorectal cancer screening in community health centers. Cancer Epidemiol Biomark Prev. 2020;29(6_Supplement_2):C123. [doi: 10.1158/1538-7755.DISP19-C123]
27. [Reference 35] Rocha HA, Dankwa-Mullan I, Meneleu P, et al. Using implementation science to examine impact of a social responsibility agenda on addressing cancer health disparities in Ceará, Brazil. J Clin Oncol. 2020;38(15_suppl):e19071. [doi: 10.1200/JCO.2020.38.15_suppl.e19071]
28. [Reference 42] Gallegos D, Do H, Alraman H, Vo B, McKechnie R. Living well multicultural - does it change behaviours in migrants to reduce the risk of chronic disease. Rev Esp Nutr Hum Diet. 2016;20(Suppl 1):521.
